# Reflecting on partnerships established and sustained over four cycles of a federally funded cancer prevention and control research program: lessons learned for community-academic networks

**DOI:** 10.3389/fpubh.2024.1384588

**Published:** 2025-01-07

**Authors:** Swann Arp Adams, Lauren Workman, Mayank Sakhuja, Brooks Yelton, Karen E. Wickersham, Ciaran Fairman, Jan Eberth, Sue Heiney, James R. Hebert, Jaron H. King, Freda Allyson Hucek, Lauren Schaurer, Daniela B. Friedman

**Affiliations:** ^1^Department of Epidemiology and Biostatistics, Arnold School of Public Health, University of South Carolina, Columbia, SC, United States; ^2^Biobehavioral Health and Nursing Science Department, College of Nursing, University of South Carolina, Columbia, SC, United States; ^3^Department of Health Services Policy and Management, Arnold School of Public Health, University of South Carolina, Columbia, SC, United States; ^4^Center for Applied Research Evaluation, Arnold School of Public Health, University of South Carolina, Columbia, SC, United States; ^5^Department of Health Promotion, Education, and Behavior, Arnold School of Public Health, University of South Carolina, Columbia, SC, United States; ^6^Department of Exercise Science, Arnold School of Public Health, University of South Carolina, Columbia, SC, United States; ^7^Department of Health Management and Policy, Dornsife School of Public Health, Drexel University, Philadelphia, PA, United States; ^8^Cancer Prevention and Control Program, Arnold School of Public Health, University of South Carolina, Columbia, SC, United States; ^9^Arnold School of Public Health, University of South Carolina, Columbia, SC, United States

**Keywords:** cancer disparities, African American, evidenced-based interventions, community-based participatory research, cancer education and communication

## Abstract

**Introduction:**

The Centers for Disease Control and Prevention (CDC) funded Cancer Prevention and Control Research Network (CPCRN) is a national network which aims to accelerate the adoption and implementation of evidence-based cancer prevention and control strategies and interventions in communities, enhance large-scale efforts to reach underserved populations and reduce their cancer-related health disparities, and develop the capacity of the dissemination and implementation work force specifically in cancer prevention and control.

**Methods:**

Our site has been a part of the CPCRN since its inception in 2002 with the exception of the 2004–2009 funding cycle. As community-based participatory research is a core value of our center, we examined the development and continued engagement of our community partners using a qualitative, inductive approach to identify emergent themes from focus group sessions with current and past investigators.

**Results:**

Several key themes were identified from our analysis including long-term commitment to community partnerships and interconnectedness with other work, authentic approach, valuing our community as experts, and mutual benefits.

**Discussion:**

With our results, we provide evidence of common community-based participatory research (CBPR) principles which have supported the sustained engagement with those racial minorities who are most vulnerable in our community. While future analysis is planned to utilize this same approach with our community partners, this work marks an important step in reflecting upon the approaches which have led to our success and how they can be applied in future collaborations to maximize impact and sustained health improvements.

## 1 Introduction

Since robust cancer statistics have been available beginning in the late 1990s, we have seen that South Carolina has consistently had cancer incidence and mortality statistics that are statistically significantly higher than the US as a whole (1). Discrepancies between state-level and national statistics are driven almost entirely by racial disparities that tend to disfavor African Americans in comparison to their European-American counterparts (2–6). Disparities in mortality tend to be much larger than those in incidence, thus acting as a multiplier that leads to extraordinarily high cancer death rates, such as we see in prostate and colorectal cancer (7–9). Over time, there has been a gradual improvement in both incidence and mortality rates for all races; however, the disparity gap between European and African American races has become wider (10). The South Carolina Statewide Cancer Prevention and Control Program (CPCP) has been funded by both the Center for Disease Control and Prevention (CDC) Cancer Prevention and Control Research Network (CPCRN) and the National Cancer Institute (NCI) funded Community Networks Program (CNP) Centers to address these disparities (11–13). Consequently, relative differences in racial rates of cancer incidence and mortality have improved slightly in South Carolina over this period of time (14).

**Figure 1:**
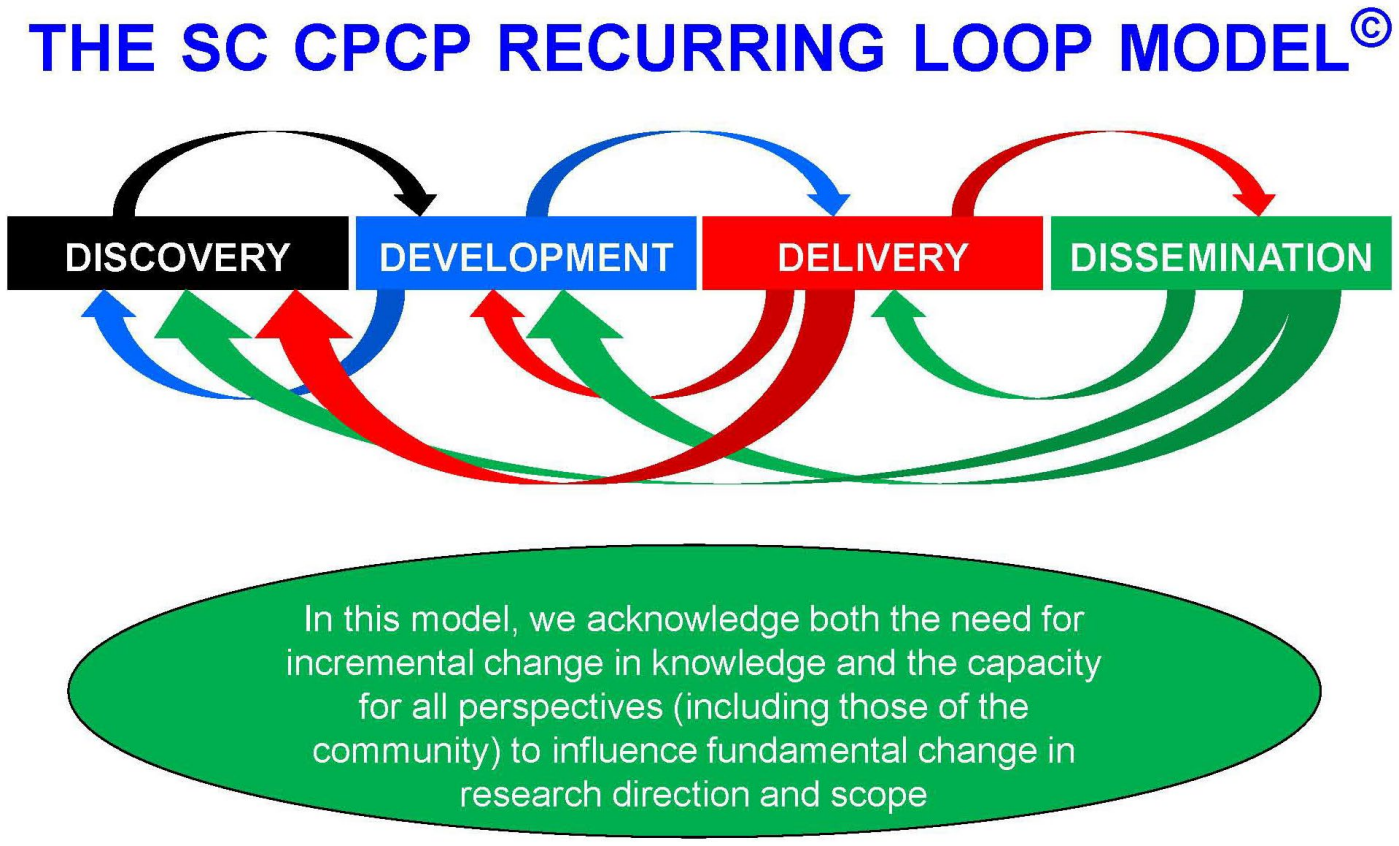
Model of scientific development in the SC CPCP.

From the inception of both the CPCP and CPCRN (then called the Cancer Research Network; CRN) in the early 2000s we have had a consistently strong commitment and focus on community-based participatory research (CBPR) to address the overall burden of cancer in South Carolina and, by logical inference and modeling, for the US as a whole (12, 15–17). In the process of conducting this work we have come to realize that community-focused efforts not only help with delivery and dissemination of successful interventions but also can inform basic discovery science. Thus, we have focused our efforts on the entire cancer research continuum from discovery to development, delivery, and dissemination (15). Recognizing that science proceeds both with incremental change and, occasionally, rapid paradigm-shifting innovation, we designed the recurring loop model (Figure 1). Not only does the model allow for depiction of the reality of how scientific innovation arises and human efforts to improve health occur by implementing and disseminating successful in innovations, but it also allows for immediate feedback from any part of the continuum to any other part. This is important for recalibrating and readjusting efforts, especially at the delivery and dissemination ends of the continuum.

Partnering and engaging with community members and organizations is an extremely effective way to improve cancer-related health outcomes and reduce health disparities (18–27). CBPR involves partnerships between academic and community organizations to conduct collaborative research on identified community needs and to translate research into practice and policy for social change and mitigating health disparities (25). Community-engaged work helps to educate people about the preventable causes associated with cancer, such tobacco use, unhealthy eating, and lack of physical activity (18, 19, 23). Interventions that involve a community partner help raise awareness about the benefits of timely screening for cancer and promotes healthy behaviors and lifestyle changes (22). A community-based randomized controlled trial, which involved collaboration with community churches, for cervical cancer screening found that participants in the treatment group that received a culturally tailored lay health advisor home visit and a newsletter that addressed barriers to screening were two times more likely than the control group to report getting screened for cervical cancer post intervention (27). Community-engaged research also addresses cancer disparities by providing preventive services to underserved populations who have limited access to quality healthcare (27). Such initiatives have implications for developing interventions for cancer prevention. Working closely with community partners helps better understand the needs and experiences of the community itself and helps develop culturally and socially relevant interventions for cancer control and prevention (24). For individuals affected by cancer, support groups and community-based cancer programs can offer assistance that targets social determinants of health needs such as transportation, financial assistance, access to mental health counseling services (20, 21, 26). Over time, the biomedical research community has struggled with recruiting minority and underrepresented population groups in advancing cancer research. However, using principles of community-based participatory research that employs involvement of community partners in all aspects of the research activities has proven to be highly effective strategy for enrolling underrepresented groups in cancer research studies (17).

The purpose of this manuscript is to focus on the important partnerships that have enhanced this network over time and demonstrate the value of such partnerships for other similar research networks with community, academic, and clinical partners. The purpose of this paper is to describe the: (1) South Carolina Cancer Prevention and Control Research Network’s (SC-CPCRN) number, diversity, and participation of partners over time, and (2) processes used to foster partnerships for cancer prevention and control and dissemination and implementation science via the South Carolina CPCRN. Multiple data sources will be used to illustrate the partnerships, including how community partnerships with the SC-CPCRN led to additional collaborations with other university, community, or state agency partners (28–36).

## 2 Methods

### 2.1 Data collection

This work draws from the case study research approach, which calls for gathering detailed information to understand an issue in-depth (37). The case study approach, applied in this work, used the research team as our unit of analysis, given our interest in understanding the team’s development of partnerships over time. For our qualitative data, a series of four focus group discussions were conducted using a semi-structured discussion guide. Participants included current and former investigators (*n* = 10) and former program managers (*n* = 2). The discussion guide included questions to understand factors that facilitated community partnerships for cancer prevention and control. In our discussion, we asked investigators to reflect back on the duration of their involvement with the research network, which in some cases, dates back to 2002. Sample questions included, “*Thinking back, tell me about some of strongest community partnerships that were generated through CPCRN*,” “*How did the principles of CBPR shape your approach?*,” and “*What are some of the most important facilitators of those relationships*?” All discussions were recorded and transcribed *verbatim* for analysis. The University of South Carolina Institutional Review Board approved this study.

For our quantitative data, we created a comprehensive listing of all community and organizational partners from our letters of support and named partnerships from our grant application materials. All current members of the SC-CPCRN investigative team reviewed this listing for accuracy. In addition, the team was asked to provide additional community partner names if not already provided in the list.

### 2.2 Analysis

Our data analysis was guided by an inductive approach wherein emergent themes were identified in the data (38–40). Qualitative data analysis software (Dedoose) was used to code data and organize the analysis (41). All focus group sessions were transcribed, assessed for accuracy, and analyzed using open coding techniques. The analysis was an iterative, multistage process that involved comparing, contrasting, coding, and reflecting on data as it was collected. All interview transcripts were open coded; during this first pass, two research team members independently reviewed a transcript. After individual review, the team compared interpretations of initial themes and emergent codes. Upon resolving any variation in interpretations, the team developed a list of themes to focus the analysis. Then, the codebook was refined and organized around those emergent themes. Finally, a comprehensive round of coding was completed with all transcripts. A finalized codebook was developed with 28 codes, which is available as a supplementary file with this manuscript (Supplementary Table S1). All participants were provided with a summary report and asked for their feedback to verify interpretations. Pseudonyms are used throughout our manuscript to protect the privacy of our participants.

Our community partnership data was compiled into broad categories in which the frequencies and proportions were calculated. All calculations were done via Microsoft Excel^®^.

## 3 Results

Table 1 provides a brief description and distribution of types of community partners identified in our network analysis. The majority of our partnerships were with organizations (50%) followed by community leaders and community mini-grantees. Of the partnering organizations, the highest proportion were general healthcare organizations (35%) followed by public health organizations (15%), cancer organizations (15%), and oncology healthcare organizations (15%; see Table 2).

**Table 1:** Types and frequencies of community partnerships developed through the South Carolina Cancer Prevention and Control Research Network, 2002–2024.

Type	*N* (%)
Community mini-grantees	9 (17%)
Organizations	26 (50%)
Community leaders	17 (33%)
Total	52

**Table 2:** Sub-types and frequencies of partnering organizations developed through the South Carolina Cancer Prevention and Control Research Network, 2002–2024.

Organization sub-type	*N* (%)
General Community Organizations	3 (12%)
General Healthcare Organizations	9 (35%)
Oncology Healthcare Organizations	4 (15%)
Cancer Organizations	4 (15%)
Public Health Organizations	4 (15%)
Faith-based Organizations	1 (4%)
Commercial Organizations	1 (4%)
Total	26

Several key themes emerged in our data, including *a long-term commitment to community partners, a focus on authentic relationship building, celebrating the expertise of community members, developing community generated solutions, and ensuring mutual benefit was derived from community participation.*

### 3.1 A long-term commitment from investigator team to community partners coupled with intentional overlap of funding

The SC-CPCRN team has an extensive history of community-based work and partnerships. A longtime SC-CPCRN Principal Investigator (PI) shared the history of those early partnerships.

*When the Cancer Research Network started, it was with the Black Baptist churches and with the faith community…working with the Woman’s Baptist Education and Missionary convention and the Young Woman’s Auxiliary …that was really the primary community partner in [that time]. Of course, there were others, like the South Carolina Cancer Alliance, the South Carolina Department of Health and Environmental Control, and other key players.*

However, the roots of community partnerships predate this funding opportunity, as the original team of lead investigators were active in community-based and professional organizations focused on cancer prevention and control. One investigator explained:

*We have a history of being community based, so our partnerships reflect that value. The community recognizes we care, and we have had a long-standing commitment.*

Over time, the project’s leadership team intentionally built a system of overlapping initiatives, which allowed partnerships to thrive. One investigator explained how they facilitated this alignment:

*Having a network gives a* var*iety of connections, and when alignment occurs, or an opportunity appeared, we would talk about it and recruit partners. The community was a catalyst for the way we work on other projects, and that led to other spin offs and collaborations.*

For example, the SC-CPCRN was not funded from 2004 to 2009. Yet, even during this time, the investigators and partners remained engaged, and the work continued because of the team’s existing work around several of the similarly oriented cancer prevention and control initiatives. For example, a “sister” project—the SC Cancer Disparities Community Network—allowed an existing space for the work to continue. One investigator, who has nearly 20 years of experience with the SC-CPCRN, shared their perspectives on this important aspect of the group’s history.

*The South Carolina Cancer Disparities Community Network, kind of filled a stopgap, because that was first funded in 2005, and that was that period of time when we did not have a CPCRN. So, that offered a vehicle to keep moving forward with the ideas and processes. So, in 2009 when we got the funding back…the way in which the project was organized at that time was really just around building on and leveraging the South Carolina Cancer Disparities Community Network. Quite honestly, that was really an important feature of the application to get back the funding*.

Also, during this time, partnerships with federally qualified health centers (FQHCs) and their statewide unifying organization, the SC Primary Healthcare Association, blossomed through a shared goal of reducing cancer disparities. One investigator explained the key partnerships during this time.

*That’s when the FQHCs came into play, in that 2009 application…and the South Carolina Cancer Alliance, the state health department…and the partnerships were with South Carolina Oncology Associates, the South Carolina Central Cancer registry, the state Office of Research and Statistics* ^[1]^ , *and the national Community Cancer Center program, which at that time was at Spartanburg Regional through the Gibbs Cancer Center…here was a broad range of different key allies and partners and the people who were involved, but really the Cancer Disparities Network kind of let that stopgap in there.*

In addition, around the same time period, several other grants were awarded to the investigator team “*that w*as *the confluence of partnerships*.” This early focus on community partnerships was investigator driven, but also supported by federal project officers which allowed the team to create a community-centered niche. For example, the SC team was the first to bring community partners to federal project and other official grant-related meetings. One investigator discussed the team’s commitment to community-engaged research during this period.

*We were contributing to a national movement, and we were viewed as leaders because of our partnerships with FQHCs. So, there was also a lot of synergy across other similarly oriented community-engaged research that was happening at that time, where we had the South Carolina Cancer Disparities Community Network, we had community health educator funding, we had the CPCRN, and many of us had individual research projects that all leveraged across these partnerships, and all shared a commonly woven thread that really weaved a fabric of connectedness and collaboration that helped contribute directly to our success.*

While funding is a critical aspect of success, the SC-CPCRN team did not parcel out program or fund specific initiatives. Rather, there was a broad and intentional approach which allowed space for partnerships to flourish and the investigator team to demonstrate their commitment to CBPR. One investigator team member explained the SC-CPCRN’s longstanding commitment to community rather than a focus on funding.

*The blurring of the lines…it wasn’t an accident, it was intentional. When we had those early grants—up until 2012—we were the only university in the country that had both a CPCRN and a community networks program under the same roof. I was the PI of both of those until Mary* *took over the CPCRN in the '09-'14 cycle. Mary and I may be different in many ways, but I think philosophically, we agreed on the intentionality of blurring the lines. We wanted to do that and…we just made that decision and brought people along…there’s been really no philosophical change in leadership; it is excellent, because we still have that same impetus that drives everything forward.*

### 3.2 An authentic approach to partnership development and relationship building

A foundational aspect of the SC-CPCRN’s long term community partnerships has been the investment of time in building relationships. Communities are often hesitant to partner with researchers because of past wrongdoings and the history of communities being misled. This history created an important orientation to how the SC-CPCRN team approached the work, as well as the need to directly acknowledge the political and historical context of living and working in South Carolina. One investigator explained:

*We had been hearing from our communities, you know, that a person shows up, they wanna do research [and] work with them to do a grant, and then they disappear. I mean over and over and over again. That was something that made people hesitant to participate. So that orientation toward building communities from the inside out was one of [our] themes*.

The team’s orientation and commitment to relationship building has focused on fostering genuine personal connections, displaying authenticity, and being dependable. One participant in the discussion shared how the team’s approach to making real connections with people has always been central.

*We all valued the input of community members and never questioned the value that they brought to the process, because we may know all of the data points and be able to talk about the etiology of a condition, but what they know is that people in their community are dying before they should… and there’s something really wrong with that. They cannot access healthy foods. They have limits on accessing health care. They’re living in parts of neighborhoods that are polluted or otherwise subject to disadvantage. That’s been perpetrated for hundreds of years. So, I think everybody just had a real genuineness to the value that partners brought, because look, it wasn’t getting done alone.*

One senior investigator explained the commitment of time and effort a team needs to build authentic community partnerships. They explained:

*Sometimes it might be a baby step process where you have a relationship with one person, and then they introduce you to another person, and then to another person… there are lots of advocates at the community level that are working hard to make a difference, but it’s not always evident who they are, particularly to a academician who lives in their own bubble, so it starts with getting out of your office and into the community, and that takes time and effort beyond 8 to 5…you are doing nights, you are doing weekends, you might be going to a funeral [or] a market…you might be going to some sort of community event…and it is at those events you often meet people, and that starts building a relationship. It takes time to establish those relationships, and many academicians are hesitant to spend that much time going out into the community to build those relationships.*

Others shared perspectives on how they worked to sustain relationships with community partners. For example, one person shared:

*I had met Penny* *many years ago doing work with women who were in prisons, and so it’s like, you might not be able to continue some of that work. But because of the genuine “you” that you brought to the table, and because of the way that you contributed and in the absence of maybe a well-funded endeavor that there was a lot of connection that continued. I love to work with her…these projects come and go, but these relationships, you want to make sure you sustain. And I think folks did that.*

### 3.3 Listening to learn: recognizing community as experts

The SC-CPCRN’s orientation toward long-term relationships with community partners has been bolstered by their recognition of the community’s expertise. For example, one community partner representing the SC Primary Health Care Association has been formally involved as an investigator in several grant cycles, beginning in 2009. The SC-CPCRN’s orientation toward listening to learn from the community can be illustrated in story mapping exercises led by SC-CPCRN investigators. One team member described how this provided a space to listen and learn from the community.

*[Health disparities story mapping] was a really powerful tool to engage with the community in an authentic way that we were listening and wanted the community’s input. That really helped a lot in beginning to cement that relationship. It’s amazing how that just really helped us solidify that authenticity, that trust that we are listening and acknowledging the expertise of the community.*

The investigator team also recognized their own lack of understanding of their community partners’ lived experiences, which required them to listen. One team member shared:

*In South Carolina, our partners were exceptional. There’s a level of readiness where it’s more than just being passionate. It’s willingness to have the hard conversations to take on barriers and challenges, to be creative when barriers cannot be overcome. They know the problems better than we could possibly ever know them, especially if we do not have lived experience in that way. That really strengthened and enhanced our science because we were able to think about every number as a person.*

### 3.4 Building work that is mutually beneficial

The SC-CPCRN team recognized the importance of building collaborative initiatives that put resources directly into local communities and addressed their perceived needs. One notable example of this is the *Right Choice, Fresh Start* farmer’s market that was implemented with a local FQHC (35, 36, 42, 43). The market began as a demonstration project for the 2009 CPCRN funding cycle and was grounded by an intensive statewide readiness assessment. Over a short time, the *Right Choice, Fresh Start* farmer’s market grew into a highly successful initiative that resulted in behavioral and state and federal policy changes. Moreover, it has been sustained because it was built as a community-led initiative, supported by a community advisory council from the beginning.

Another innovative, community-oriented initiative that grew out of the SC-CPCRN was the community health improvement program (CHIP) mini-grant initiative. This has allowed communities to have direct access to SC-CPCRN funding to implement a small program that addressed an important community-identified local need. One investigator shared their perspective on the importance of providing a small, but impactful financial investment into communities.

*The CHIP program mini grants that were given out really extended the work. One of the challenges still today…is how you share these resources in a very meaningful way. True, authentic CBPR talks about 50/50, right? Sharing with external partners in practice is incredibly complicated, and so how do you overcome that? You overcome that because you could connect with people, you know what they like. You meet them on their turf. You go to them, you find ways to make investments…and then you watch the CHIP program…What happens when you really do give folks money and get out of the way? It’s magical to watch when you have a system set up to invest in people, build capacity, and just get out of the way. And what community partners did with $5,000—not very much money—[was amazing].*

Another former project coordinator echoed the importance of the CHIP mini-grants, especially how the SC-CPRCN team approached the partnership. They explained:

*In some ways, I feel like the community grants really was one of the biggest facilitators, I think for a* var*iety of reasons; but, most notably, that even though it’s limited resources, we are trying to funnel resources out to community partners. That money speaks a lot. Maybe, in some sense, we have also partnered it with really close relationships if you will. It’s not like a supervision, it’s more of a “let me help you, how can I help do your work?” kind of approach.*

### 3.5 Team value for community collaboration and mutual benefit over academic products

The SC-CPCRN team has published numerous scholarly articles and is a highly productive group. However, the traditional metrics of academic productivity have not driven the SC-CPCRN’s sense of purpose. A number of team members discussed the ways in which their value for social justice and health equity drives their passion for and approach to community based work. A team member explained:

*Something that makes this work are people who are willing to find a way to move forward together and collectively for better good—people who are willing to figure out how to find common ground, and compromise, and not worry about the money, the papers, the academic pressures and products. There’s a higher sense of purpose and a higher calling in the people who have been involved of the CPCRN. We did generate products, it was a very productive group, but never at the cost of relationships with our community partners and progress with our partners.*

In addition, many junior team members reflected on how senior investigators modeled collaboration within the team and with community partners. According to them, senior investigators recognized the importance of a team with a range of skills and expertise (now known as team science) and they considered community members as investigators.

This culture of collaboration and purposeful dissemination spread to partners collaborating in academic activities like manuscript writing, as well as investigators and project staff attending important community events. Attending these community events are an important dissemination activity for the SC-CPCRN team, because they are not only a way to celebrate with partners, but also honor their work and contribution. For example, one investigator shared their experience attending a local church’s celebration.

*We may be the only ones who have a mini-grant manuscript in a time capsule…with First Methodist. When the manuscript was published, we had to do an online celebration, and the City of Columbia declared it Reverend Jones’* *Day, and we had this big online celebration which was amazing. Jodie* *and others spoke, and then as part of that celebration, they put the manuscript as part of the time capsule. I do not know how many years from now they are planning to dig it up.*

The SC-CPCRN team has also used other creative means to share their work in ways that a range of people could access, including a documentary film and a toolkit on the *Right Choice, Fresh Start* farmer’s market.

## 4 Discussion

With this investigation using an inductive, qualitative analytic approach, we aimed to describe our collaborative partnerships within our research network and identify strategies which enhanced these collaborations. We identified several key themes from our qualitative analysis of our investigator team (both past and present) including long-term commitment to community partnerships and interconnectedness with other work, authentic approach, valuing our community as experts, and mutual benefits. We firmly believe that these principals played highly into improving the impact of our work in cancer prevention and control across our state. While this study is an important component to our analysis of our community partnerships, we also acknowledge that a key next step is conducting a qualitative study with our community partners. Hence, additional study is necessary.

We are not surprised to find that valuing our community as experts emerged as a theme. Given that community members are contextual experts regarding their needs and preferences for information and resources, it is critical for researchers to collaborate with and engage them as partners in cancer prevention and control education efforts. The long-term community collaborations enhance community resilience, encourage sharing of information and resources with communities to improve health outcomes, and facilitate their engagement in program and policy development and decision-making (17, 44, 45). Evaluations from our mini-grants program clearly indicate that communities empowered to lead and implement evidenced-based strategies and programs have experienced healthier diets, increased physical activity and use of walking trails, improved cancer screening, and increased intentions to be screened for cancer following program implementation (30, 34, 46). Thus, we have direct evidence of how this community of experts resulted in increased impact on cancer prevention and control. Very early in the process of developing partnerships, the SC-CPCRN team acknowledged the importance of truly listening to the community members and making every effort to understand their experience and empathizing with difficulties they might be experiencing. Even if we thought that we understood, we often only partially understood the situation. Some examples of this co-learning process were participating in their organizational activities as well as providing technical support for a desired activity, e.g., procuring an oncology expert on a particular topic. We made efforts to celebrate our partnerships and join their celebrations of accomplishments. Giving our partners opportunities to present a summary of their accomplishments at professional meetings was indicative of our desire to let them take credit for all their hard work and reinforce our desire to listen. We also participated in community events with partners which were not an expressed part of our research work; however, we knew that it was important to our community partners that we were present and supportive of their initiatives. In this way, we created ‘shared memories’, which deepened the trust and respect of our relationship.

Related to our theme of long-term commitment, authentic approach, and mutual benefit was acknowledgement of the concept of “data rape” of the community. The issue was brought forward by a minority investigator of the team, speaking on behalf of the community early in our history as a network. The concept was described as entering into a relationship with the community in order to collect data for a specific research study, and then a feeling of abandonment within the community when the research study ended and there was no follow-up by the research team. This informed all our interactions, often without investigators being consciously aware.

Another aspect to the sustainability of the partnership was attempts by investigators to incorporate these community partnerships into other aspects of their research portfolios. This was especially important for years in which there were gaps in funding with the SC-CPCRN. There were several additional research projects which were conducted in partnership with our community that were outside the scope of the original network, but yet are important building blocks to support the partnership.

There are some limitations to our study which should be considered. As noted previously, we are presenting only the investigator perspective in our partnership analysis. We intend to expand upon this work by conducting a similar study among our community partners. Additionally, our findings present investigator perspectives on community partner engagement from one state and therefore, cannot be generalized. While our methodology provides rich data from our current and former investigative team, it is also dependent on the interpretation of the researchers. Therefore, completely eliminating the bias inherent to this process is unlikely. Efforts to add rigor to our methods included sharing results with participants for their verification and feedback, as well as having a second research team member analyze data to compare interpretations.

In conclusion, our analysis demonstrates many commonalities with current principles of community-based participatory research approaches including respect for the community and mutual benefit. Additionally, we have described practices which have served to enhance the sustainability of our partnerships as well as train the next generation of community-engaged researchers. These partnerships are an important component to realizing sustained changes aimed at improving health disparities experienced by minoritized and vulnerable populations.

## Data Availability

The original contributions presented in the study are included in the article/Supplementary material, further inquiries can be directed to the corresponding author.
