# Supplementary material for: Reflecting on partnerships established and sustained over four cycles of a federally funded cancer prevention and control research program: lessons learned for community-academic networks
Source: Front Public Health. 2025 Jan 7;12:1384588. doi: 10.3389/fpubh.2024.1384588 (PMC11747125; doi:10.3389/fpubh.2024.1384588)
Supplement: Supplementary file 1 [file Data_Sheet_1.docx]

## **Figure**


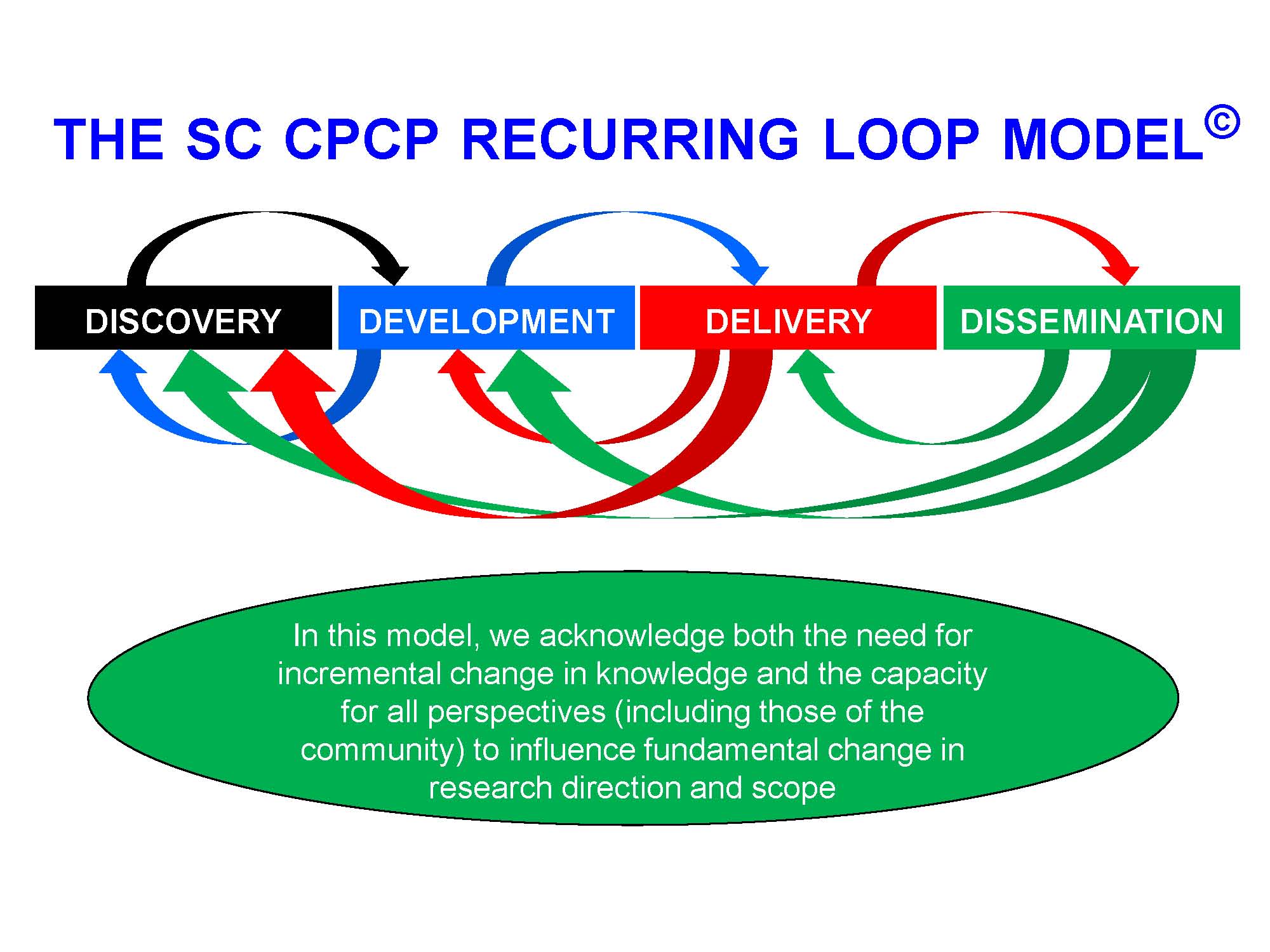


Figure 1. Model of scientific development in the SC CPCP.

**Supplementary Table**

Supplementary Table 1. Code Book.

| **Code** | **Definition** | **Example Quote** |
| --- | --- | --- |
| All the grants blurred together | Explanation of how the team focused on outcomes, rather than a specific funding mechanism | "So, there was also a lot of synergy across other similarly oriented community engaged research that was happening at that time. We had [multiple grants] and individual research projects that all leveraged across these partnerships, and all shared a commonly woven thread that really weaved a fabric of connectedness and collaboration that I really feel helped contribute directly to our to our success." |
| Health disparities story mapping | A community engaged research strategy used by the team to understand existing health disparities research | "We mapped out different areas related to health disparities, related to research." |
| Traditional research methods don't work in communities | Explanation of how traditional research designs and methods are not always well suited for community engaged research | "[A team member] authored that paper...about why traditional research methodologies don't work in communities. I feel like that that's been a huge...[it] has informed a lot of what I do...that it's just not ethically right anymore to have people do lifestyle intervention, but then not to have anything else and deny it to the other people." |
| Bringing resources into communities | Examples of how the team helped connect communities to funds and other resources for their community | "But those two developments were really important in ways that the community could really understand, because it brought serious resources into the community, like hundreds of thousands of dollars of resources which was greatly appreciated." |
| CHIP mini grants | Description of mini grant program for communities to write proposals to obtain funding for community generated solutions | "But those two developments were really important in ways that the community could really understand, because it brought serious resources into the community, like hundreds of thousands of dollars of resources which was greatly appreciated." |
| Community clinical linkages | Building partnerships with clinical and healthcare partners in the community | "It had a very tangible benefit...all of the projects that I've ever done have a little bit more of that community angle...we're going to be working with community-based hospitals." |
| Community focused/community based | Team orientation to integrating community member's input into the work | "That's been foundational...[one of our research team leaders] was fascinated and interested by the expertise and contributions that community members brought to the table and was very open and supportive…and he really was very committed to that. " |
| Community impact | Reflections on how the work has made a difference in partnering communities | "One of the unique things about the CPCRN is that we can see the impact more immediately. There's a great sense of satisfaction that what you're doing matters at a local level and that they do value it." |
| Community advisory board | Groups of community members who provided input on the direction of programming. | "We also had a very community engaged approach. We've had an Advisory Council related to the market." |
| Community dissemination | Strategies to share what was learned with the community | "We've been pretty good at the dissemination beyond the journals and going to conferences because that's really important to us. We've had one pagers, we've had policy briefs, story maps and webinars. |
| Farmer's market | A community engaged demonstration project carried out by the research team | "I'm really proud that they still have a farmers market…not only did we learn a lot, but they adopted this intervention, and they made it their own." |
| Federal influences | Discussion around federal support for community engaged approaches | "We had a program officer from the NCI who was very hands on. She came down and visited us, I toured around in my car." |
| Follow through | Ideas about the importance of doing what you say in community work | "I feel like the other thing is just that idea of credibility and showing up and doing what you're say you're gonna do and following through." |
| History of CPCRN | Genesis of the program, research team, and its partners | "We decided very early on we needed to be community focused, and we pushed right from the beginning of time to make it very CPCRN-ish. It's very gratifying to see that five [grant] cycles later." |
| Investigator passion for community-based work and social justice | Individual and team value for this type of work | "We all valued the input of community members and never questioned the value that they brought to the process because we may know all of the numbers be able to give all sorts of statistics, but what they know is that people in their community are dying before they should and there's something really wrong with that." |
| Investigator team modeling CBPR/mentoring | Reflections on how senior team members mentored junior members | "Many of the other people who were involved were already relatively established, but this was incredibly important to my growth and development and ability to interact and network with leaders in the field and also peers in the field who were interested in doing this type of research well also within our university setting." |
| Key community partners | Individuals and organizations who collaborated with the research team | "The foundational work with federally qualified health centers to understand to understand how to better do research with them and find ways to align our efforts with them." |
| Lapse in funding | Discussion around a cycle in which the research team did not receive the funding award | "And so that was that period of time when we did not have a cancer prevention and control research network." |
| Listening and acknowledging the expertise of the community | The research team's value for community knowledge and experience | "By going to the community…and doing something with them. You had to start doing things with them to show that you were gonna do something and listening to them and doing it." |
| Manuscripts | Academic products to disseminate lessons learned and key findings | "The way that we're gonna shift the field is as researchers is to get the evidence from this kind of research into the scientific literature and if we don't, then they're just stories that we told in our community." |
| Mutual benefit | Ideas about how research should be conducted to benefit community and researchers | "They've continually engaged with the same church, which shows to me that, it's more than just about getting [money], it's about like this sustained community relationship that they've built that they continue to come back and want to work together is a real testament to how those relationships have been very positive for both parties." |
| Not a cancer center CPCRN | Distinguishing ideas about value of a community-based research network | "I think we're the only non-designated cancer center, cancer group working within this larger network. It's been very thoughtful about how do we create our niche...we were always the first center to do something with a community." |
| Relationship building | Conversation about relationships and trust as the basis for partnerships | "Something that makes this work are people who are willing to find a way to move forward together and collectively for better... people who are willing to not try to force square squares through round holes and willing to figure out how to find common ground and compromise." |
| Role with CPCRN | Individual's role within the team | "It was 2020 [when I] joined CPCRN... I'm a fairly new member, but an enthusiastic one." |
| Sustainability of partnerships | Continued connections and partnerships beyond life cycles of grants | "So, I think the network really is pretty special, I could still probably pick up the phone right now and reach out to [partner].". |
| Tangible impacts | Clear results from the work done | "You could actually see the work that we were doing in terms of improving cancer related outcomes over those periods of time...those were really important developments that had a huge impact on the community, both in terms of health outcomes and in terms of economic development." |
| Policy change | Examples of policy changes resulting from work done in partnership with communities | "Our work in South Carolina was fundamental to that policy." |
| University commitment to community work | Institution's value for the work | "I had time from my university to be out there…it I felt like what I was doing was well respected by my institution." |
